# Characterising the role and scope of Community Connectors for Older Adults Internationally: A Scoping Review Protocol

**DOI:** 10.12688/hrbopenres.14268.1

**Published:** 2025-10-29

**Authors:** Danielle Manning, Christina Hayes, Rose Galvin, Frank Houghton, Jennifer Moran Stritch

**Affiliations:** 1 Technological University of the Shannon Midlands Midwest, Limerick, County Limerick, Ireland; 2 University of Limerick, Limerick, County Limerick, Ireland

**Keywords:** Non-medical interventions, community-based care, social prescribing, older adults, ageing, scoping review

## Abstract

**Background:**

As global populations age, there is an increasing need for community-based approaches that support older adults in maintaining independence, social connectedness, and access to services. One emerging model is the Community Connector (CC), an individual who facilitates older adults’ engagement with local supports that promote well-being. Despite growing references to this role in national and international practice, there is limited comprehensive understanding of how CC’s are defined, implemented, and evaluated across diverse contexts, and how these insights may inform adaptation within varying health and social care systems.

**Objectives:**

The objectives of the scoping review are to (1) identify and characterise international models of CC roles and their key interventions, including modes of delivery (2) describe key characteristics of populations they serve and (3) summarise reported outcomes.

**Methods:**

This scoping review will follow the methodological framework of Arksey and O’Malley (2005) and will be in accordance with the PRISMA-ScR guidelines. The study design was chosen due to its capacity to comprehensively map and synthesise the existing literature on international CC models, particularly given the limited nature of the evidence. A comprehensive search will be conducted from 2000 to present, across academic databases [CINAHL, Cochrane Library, EBSCO, Embase, PubMed, OVID, Scopus] and grey literature to identify CC’s models targeting older adults. Two reviewers will independently screen studies for inclusion based on the criteria of full-text, qualitative, quantitative and mixed-methods studies. Data on programme characteristics, target populations, measured outcomes and contextual factors will be extracted, charted, and synthesised using a narrative approach.

**Conclusions:**

The review will characterise international community connector models, including their core functions, terminology, target population and implementation strategies. It will also examine evaluation methods and assess their potential for adaptation and scale-up across healthcare settings.

**Scoping Review Registration:**

OSF https://doi.org/10.17605/OSF.IO/6CZD8

## Introduction

The global population is ageing at an unprecedented rate due to a combination of declining fertility rates and increasing life expectancy. According to the World Health Organisation approximately 1.4 billion of the population are currently over the age of 60 years, which is set to double to 2.1 billion by 2050 ( United Nations, 2019; WHO, 2022). This demographic shift is primarily driven by reduced birth rates, improved healthcare, and advancements in disease prevention, leading to longer life spans ( Christensen *et al.*, 2009). While increased longevity is a sign of medical and social progress, it is also accompanied by a rise in multi-morbidity, defined as the co-existence of two or more chronic conditions, which poses significant challenges to healthcare systems and social care systems worldwide ( Marengoni *et al.*, 2011). Ageing with multiple morbidities presents substantial challenges such as increased healthcare needs, higher risk of disability, cognitive decline, reduced quality of life, and heightened mortality risk ( Barnett *et al.*, 2012).

With rising healthcare costs ( Hayes *et al.*, 2024; Trépel *et al.*, 2024) service providers have increasingly sought ways to better support older adults with complex needs ( Burke *et al.*, 2018; Demiris *et al.*, 2020; Government of Ireland, 1988). In doing so, there has been a growing recognition that traditional healthcare models fail to address the broader determinants of health, such as social support, housing, nutrition and access to community resources ( Kimberlee, 2015; Kingsfund, 2017; Polley *et al.*, 2017). These factors play a critical role in shaping health outcomes and overall well-being yet historically, these have been overlooked in favour of a disease-centred approach ( Giebel *et al.*, 2022; Williams *et al.*, 2023). As a result, many older adults experience a poorer quality of life despite having medical care, as their social and environmental needs remain unmet ( Husk *et al.*, 2019). To address this gap, there has been a shift towards more holistic and integrated care models that emphasize person-centred approaches, social prescribing, and community-based interventions ( Chatterjee *et al.*, 2018; Pescheny *et al.*, 2020).

Social prescribing connects individuals with complex needs to community-based non-medical support services ( Bickerdike *et al.*, 2017; Kiely *et al.*, 2022). The community connector model, an operational model of social prescribing, is grounded in the principles of holistic and person-centred care ( Polley *et al.*, 2017). The Community Connector model recognises that medical care alone is insufficient for improving quality of life and that community-based interventions are essential in promoting well-being and independence among the older population ( Giebel *et al.*, 2022; Littleford & Kralik, 2010). A community connector is a non-medical professional who is typically based in the community setting. Typically, they address wider social determinants of health through non-medical interventions and the development of an individualised and collaborative health and well-being plan, while offering support to connect with community services ( Wilson *et al.*, 2025). Preliminary evidence has suggested that the community connector initiative has the potential to address broader determinants of health by enhancing social connections, improving mental and physical health and increasing utilisation of health services ( Prendergast & Alexander, 2023; Wallace *et al.*, 2020). However, despite its potential benefits, significant barriers exist due to the lack of a standardised approach in implementing such models.

While community connectors share the common goal of addressing social determinants of health, there are key differences in how they operate globally. A significant challenge is the lack of a clear definition due to variations in their structure and implementation ( Kiely *et al.*, 2022). For instance, in the United Kingdom they are known as link workers formally embedded in the National Health Service ( NHS, 2019), while in Australia ( Larson *et al.*, 2024; Sharman *et al.*, 2022) and New Zealand ( Boulton *et al.*, 2009), they are called community navigators, focusing on culturally appropriate care for Indigenous populations. In Canada ( CPHA, 2022) and the U.S., they are referred to as community health workers, primarily supporting marginalized groups ( Larson *et al.*, 2024). In Ireland, community connectors function within integrated care programs, assisting older adults with complex social and health needs ( Hannan & Sieger-Jamison, 2024). Thus, without clear guidelines or a unified framework, there is considerable variation in how these services are delivered, leading to inconsistencies in their effectiveness. This was emphasised in a recent systematic review conducted by Kiely *et al.* ( Kiely *et al.*, 2022), which examined evidence from randomised controlled trials and controlled before-after studies to determine the effectiveness of social prescribing link workers. Findings from this review demonstrated significant heterogeneity in intervention design, populations served, and outcome measures across, leading to inconsistent evidence regarding the impact of social prescribing on health outcomes and healthcare utilisation ( Kiely *et al.*, 2022). Furthermore, authors of this review called for future research to better understand the components of social prescribing and outcomes considered for older adults ( Kiely *et al.*, 2022).

### Aims

The scoping review aims to identify and characterise international models of community connector roles for older adults, including their key interventions and modes of delivery; to describe the populations they serve, and to summarise the reported outcomes associated with these interventions. This review addresses a key gap in the literature by examining community connector models across acute, primary, and community care settings to provide a comprehensive understanding of their roles in diverse healthcare contexts.

***Objectives***

The objectives of the scoping review are to (1) identify and characterise international models of CC roles and their key interventions, including modes of delivery (2) describe key characteristics of populations they serve and (3) summarise reported outcomes.

## Methods

A scoping review design has been selected due to its suitability for mapping the breadth of existing literature on international community connector models, roles, interventions, and outcomes, particularly given the limited nature of the evidence and the emerging status of the field. The proposed scoping review will be conducted in accordance with the Arksey and O’Malley ( Arksey & O'Malley, 2005) scoping review framework and later refined by Levac *et al.* ( Levac *et al.*, 2010) and the Joanna Briggs Institute. The review will also adhere to the PRISMA-ScR (Preferred Reporting Items for Systematic Reviews and Meta-Analyses extension for Scoping Reviews) checklist to ensure transparency and rigour in reporting. The scoping review will be registered with Open Science Framework (OSF) and any changes to the proposed protocol will be amended with justifications provided.

## Eligibility criteria

Studies will be included if they describe, evaluate or conceptualise Community Connector models (including equivalent roles) that aim to support older adults (aged 60 years and older) in accessing community-based health or social care services. Eligible sources may include both empirical and theoretical work, with no restrictions on study design; qualitative, quantitative and mixed-methods studies will be considered.

## Information sources

A comprehensive search will be conducted across multiple electronic databases including CINAHL, Cochrane Library, EBSCO, Embase, PubMed, OVID and Scopus to identify relevant peer-reviewed literature.

In addition to academic databases, a grey literature search will be conducted in the following databases: grey literature sources (DART-Europe E-theses portal, Open Grey, Grey Literature Report (New York Academy of Medicine): www.greylit.org/, Agency for Healthcare Research and Quality (AHRQ): www.ahrq.gov/, Joanna Briggs Institute: joannabriggs.org and National Institute for Health and Care Excellence (NICE): www.nice.org.uk/, and Trip Medical database) and Trial registries including the WHO International Clinical Trials Registry Platform (ICTRP): www.who.int/ictrp/en/ will be searched. The reference list of included sources of evidence in the review will be searched for additional sources.

## Search strategy

Search terms will be adapted to the indexing of each database using Boolean operators. (AND, OR) to combine concepts. Search terms will incorporate both keywords and controlled vocabulary (subject headings) relevant to the PCC framework (Population, Concept, Context). They will be tailored to the indexing system of each specific database. Keywords will be derived from the titles and abstracts of pertinent articles and will contribute to the development of the final search strategy, alongside database-specific subject headings aligned with the PCC elements.

An example search strategy for EBSCO is as follows:

- TI ('aged' OR 'elderly' OR 'elderly' OR 'older adult*' OR 'older population*' OR 'geriatric*' OR 'senior*' OR 'elder*' OR 'old aged*' OR 'older person*') OR AB ('aged' OR 'elderly' OR 'elderly' OR 'older adult*' OR 'older population*' OR 'geriatric*' OR 'senior*' OR 'elder*' OR 'old aged*' OR 'older person*')
  AND
- (social prescribing OR TI ('social prescribing*' OR 'community connector' OR 'lay person' OR 'linkworker' OR 'link-worker' OR 'link worker' OR 'community health worker' OR 'community health facilitator' OR 'patient navigator'/exp OR 'patient navigator' OR 'well-being programme') OR AB ('social prescribing*' OR 'community connector' OR 'lay person' OR 'linkworker' OR 'link-worker' OR 'link worker' OR 'community health worker' OR 'community health facilitator' OR 'patient navigator' OR 'well-being programme').

These will be adapted according to the indexing of each database and logged in a master search strategy form using Microsoft Word. In accordance with replicability, a descriptive account of each search strategy of the databases will be provided in the appendix.

## Selection of sources of evidence

A comprehensive scoping review of the existing literature will be conducted to map the current state of knowledge related to the international role and scope of community connectors for older adults. The review will include peer-reviewed articles, systematic reviews and grey literature published since 2000.

## Data charting process

A standardised data charting form will be developed using Microsoft Excel, based on the Joanna Briggs Institute (JBI) data extraction tool for scoping reviews ( Peters *et al.*, 2020). The form will be piloted by two independent reviewers (DM and CH) on a sample of studies to ensure clarity and relevance and will be refined as needed. Following piloting, data will be charted by one reviewer (DM) and cross-checked by a second reviewer (CH) to ensure accuracy and consistency. Any discrepancies identified during this process will be resolved through discussion, and if consensus cannot be reached, a third review (RG) will be consulted to adjudicate.

Assessment of the risk of bias or methodological quality of included studies is not relevant as the aim of this scoping review is to characterise international models of community connector roles for older adults ( Peters *et al.*, 2020).

## Data items

The following data items will be extracted from each included source:

- author(s)
- year of publication, and country of study
- study design and methodology
- name or description of the Community Connector model; setting in which the model was implemented (e.g., community, primary care, social services)
- target population characteristics (e.g., age group, health or social needs); details of implementation processes
- outcomes measured and indicators used
- and information on funding sources or declarations of interest.

These items will support a comprehensive synthesis of how Community Connector models are conceptualised, applied, and evaluated across diverse contexts.

## Synthesis of results

The synthesis of results will be guided by the Population-Concept-Context (PCC) framework, as recommended by Joanna Briggs Institute for scoping reviews ( Peters *et al.*, 2020). Extracted data will be organised to capture key characteristics across included studies, including the target population, Community Connector model components, implementation strategies, outcomes measured, and contextual factors. A descriptive numerical summary will be used to map the distribution of studies by variables such as publication year, country, setting, study design, and population characteristics.

Qualitative data will be analysed narratively (i.e., narrative descriptions of roles, implementation processes, and experiences) to identify common patterns, functions and challenges associated with Community Connector models. A basic coding framework will be developed to categorise emerging themes. Quantitative data will undergo basic descriptive analysis to summarise variables such as publication year, country of study, and population characteristics. Findings will be presented in narrative form and supported by tables and visual mappings where appropriate to enable interpretation and knowledge dissemination.

## Dissemination plans

Findings will be disseminated through publication in a peer-reviewed open-access journal, conference presentations and target summaries for practitioners. Where possible, public-facing materials will be developed to enhance accessibility and knowledge distribution.

## Ethics and dissemination

Not relevant.

## Discussion

This scoping review will provide a comprehensive overview of international community connector models supporting older adults, highlighting variations in strategies, outcomes and contextual influences. It will provide critical insights into how these models can be adapted to other health and social care landscapes.

## Data Availability

No data are associated with this protocol. Open Science Framework: Characterising the Role and Scope of Community Connectors for Older Adults Internationally: A Scoping Review Protocol. Available at: https://osf.io/6czd8/overview. DOI: 10.17605/OSF.IO/6CZD8.
