# Supplementary material for: Characterising the role and scope of Community Connectors for Older Adults Internationally: A Scoping Review Protocol
Source: HRB Open Res. 2026 Apr 16;8:117. Originally published 2025 Oct 29. [Version 2] doi: 10.12688/hrbopenres.14268.2 (PMC13032101; doi:10.12688/hrbopenres.14268.2)
Supplement: Peer review history

# Reviewer report: Reviewer response for version 2

**DOI:** 10.21956/hrbopenres.15872.r54704

**Published:** 2026-05-05

**Authors:** Megan O'Grady

**Affiliations:** 1 University of South Carolina School of Medicine Greenville Campus (Ringgold ID: 368074), Greenville, South Carolina, USA

I am satisfied that the methodology is now described in more detail. However, the authors still have not defined their Population-Concept-Context criteria in the context of this scoping review. The PCC not only guides synthesis of results but should also be used to define the review question so that readers have a clear understanding of the scope of the review. Similarly, the authors still have justified their claim of "limited evidence" and whether an initial scoping search of the literature was performed.  Please consider these points for future publications or dissemination of the results of the review.

Is the study design appropriate for the research question?

Yes

Is the rationale for, and objectives of, the study clearly described?

Yes

Are sufficient details of the methods provided to allow replication by others?

Partly

Are the datasets clearly presented in a useable and accessible format?

Not applicable

Reviewer Expertise:

Social prescribing, scoping reviews, health promotion, physical activity

I confirm that I have read this submission and believe that I have an appropriate level of expertise to confirm that it is of an acceptable scientific standard.

# Reviewer report: Reviewer response for version 1

**DOI:** 10.21956/hrbopenres.15695.r53427

**Published:** 2026-03-27

**Authors:** Patricia M Darcy

**Affiliations:** 1 University of York, York, UK

Thank you for the opportunity to review this work. This review aims to synthesise the international literature on “community connectors” in older adults, and I commend the authors for addressing this important area.

 My comments focus on clarifying how the “community connector” model aligns with or differs from the broader social prescribing model, in which a “connector” typically refers individuals to a range of local, non-clinical services. I also recommend more clearly defining the overall focus - specifically, whether the review examines the “community connector” as an operational model or as an individual role, and ensuring consistent use of this terminology to reflect the review focus throughout the manuscript.

 1. Abstract: “One emerging model is the Community Connector (CC), an individual who facilitates older adults’ engagement with local supports that promote well-being. Despite growing references to this role in national and international practice…..” The opening lines of the abstract refer to the community connector as both a model and an individual. Please clarify whether the ‘community connector’ in this review refers to an individual role or as an operational model, and ensure consistent terminology is used throughout the manuscript.

 2. In the abstract, the authors note that “there is limited comprehensive understanding of how CCs are defined, implemented, and evaluated across diverse contexts, and how these insights may inform adaptation within varying health and social care systems.” Given the limited clarity around how a community connector is defined, should the review consider including conceptual mapping of community connectors as a key aim. It is also not clear whether ‘CC’ in this instance refers to an individual role or as an operational model, and this should be clarified.

          3. Abstract: The authors note that “the objectives of the scoping review are to (1) identify and characterise international models of CC roles and their key interventions, including modes of delivery; (2) describe key characteristics of populations they serve…”. Please clarify what is meant by “international models of CC roles” - does this refer to the roles of individual community connectors, or to an operational model. The authors could include ‘older adult populations’ under objective (2).

 4. Abstract: “Data on programme characteristics, target populations, measured outcomes and contextual factors will be extracted, charted, and synthesised using a narrative approach”. Should the data charting also capture how the community connector is defined (as an individual role)? This would align with the authors’ statement that “there is limited comprehensive understanding of how CCs are defined.”

 5. Abstract: “The review will characterise international community connector models, including their core functions, terminology, target populations, and implementation strategies, as well as examine evaluation methods and assess their potential for adaptation and scale-up across healthcare settings”. As noted previously, it is unclear whether the review focus is on the community connector as an operational model, as an individual role, or both – please clarify and ensure consistency throughout. Additionally, please clarify what is meant by “terminology” in this context.

 6. Introduction (paragraph 2): It would be helpful to more clearly define what is meant by “environmental needs” in this context, and to provide examples relevant to the population under study to enhance clarity for the reader.

 7. Introduction: In the paragraph starting “With rising healthcare costs (Hayes et al., 2024; Trépel et al., 2024) service providers have increasingly sought ways to better support older adults with complex needs” …. This point could be strengthened by more explicitly engaging with the intersection between population ageing, increasing multimorbidity, and existing pressures on health and social care systems. Incorporating relevant evidence and references would help to situate this argument within a broader demographic and policy context, particularly in relation to the growing complexity of care needs and the implications for service demand, integration, and sustainability.

 8. The manuscript may benefit from further nuance in its discussion of social prescribing. Social prescribing typically targets individuals with ‘non-medical’ needs and may not always be appropriate for, or inclusive of, those with more complex mental or physical health needs. Acknowledging this would strengthen the conceptual clarity of the paper. See for example, Cartwright et al (2022) https://socialprescribingacademy.org.uk/media/jaibqf4q/evidence-review-who-is-accessing-social-prescribing.pdf

 9. Can the authors please expand on and clarify the sentence “The community connector model, an operational model of social prescribing” to help readers understand the distinctions between the community connector model and the social prescribing model, as well as the practical implications. See Muhl et al. (2023), who discuss internationally accepted conceptual and operational definitions of social prescribing (BMJ Open 2023;13:e070184, doi:10.1136/bmjopen-2022-070184).

 10. Related to this comment, could the authors make explicit how the ‘community connector model’ differs from a ‘social prescribing model’ as this is not sufficiently clear.

 11. The manuscript states that link workers are formally embedded within the NHS. It may be helpful to add that social prescribing link workers are also employed in VCSE organisations in the UK. See Tierney et al (2025) https://doi.org/10.3399/BJGP.2024.0279 and Hazeldine et al (2025) https://doi.org/10.1136/bmjph-2024-000941

 12. The manuscript defines a community connector as “a non-medical professional who is typically based in the community setting”. Could the authors clarify if community connectors are non-clinical individuals operating both within and outside social prescribing pathways? If so, please make this explicit and provide supporting references.

 13. The authors note that “This review addresses a key gap in the literature by examining community connector models across acute, primary, and community care settings to provide a comprehensive understanding of their roles in diverse healthcare contexts,” and under “Selection of Sources of Evidence,” they state that a “comprehensive scoping review of the existing literature will be conducted to map the current state of knowledge related to the international role and scope of community connectors for older adults.” Please review the manuscript to ensure consistent language, so it is clear throughout whether the focus of this review is on the community connector as an individual role or as an operational model, or both.

 14. Methods: Have the authors undertaken preliminary scoping searches to support their claim of “the limited nature of the evidence”? The basis for this justification is currently unclear.

 15. Eligibility Criteria: Are there any exclusion criteria or limitations applied?

 16. Search Strategy: The review adopts a PCC framework; however, it is unclear how “context” has been operationalised, within the search strategy. If context is a key component of the review question, please clarify whether and how it has been incorporated into the search, or justify its omission.

 17. Selection of Sources of Evidence: The authors restrict included studies to those published from 2020 onward. Please provide a clear rationale for this limitation, explaining how it aligns with the objectives of the review and whether earlier studies may have been relevant.

 18. General: The terms ‘Community Connector’, ‘CC’, and ‘community connector’ are used inconsistently throughout the manuscript. Please ensure consistent terminology / formatting. Similarly, models of community connector roles and community connector models are used interchangeably. Please ensure consistency of language and terminology throughout.

 19. General Context: Please consider prior reviews in the area, including the scoping review by Mulholland et al. (2025)  (https://doi.org/10.1155/hsc/4394123) and provide context how the proposed review differs from or adds to the existing literature in this area. This will help to clarify the unique contribution and added value of the current review.

Is the study design appropriate for the research question?

Yes

Is the rationale for, and objectives of, the study clearly described?

Partly

Are sufficient details of the methods provided to allow replication by others?

Partly

Are the datasets clearly presented in a useable and accessible format?

Not applicable

Reviewer Expertise:

social prescribing, community-based assets, applied health research, mental health and multi-morbidity

I confirm that I have read this submission and believe that I have an appropriate level of expertise to confirm that it is of an acceptable scientific standard, however I have significant reservations, as outlined above.

# Reviewer report: Reviewer response for version 1

**DOI:** 10.21956/hrbopenres.15695.r53424

**Published:** 2026-02-24

**Authors:** Megan O'Grady

**Affiliations:** 1 University of South Carolina School of Medicine Greenville Campus (Ringgold ID: 368074), Greenville, South Carolina, USA

Thank you for the opportunity to review this work. This scoping review will provide a synthesis of evidence regarding 'community connector' interventions for older adults and will provide an important overview of these approaches. This review is timely given the importance of community-based approaches to health, and the need to address social isolation and social health in older adults. There were some details missing in relation to the methods, but I am confident that these can be addressed by the authors.

***General comments:***

 1. You mention the Population-Concept-Context framework, and state that this will guide selection of search terms and synthesis of results. However, you do not define what these are in the context of your review. Please provide a definition of PCC. 

 2. You state throughout the methods that you will examine implementation processes and strategies of the different CC models but your objectives state that you only plan to characterise these models. Perhaps this objective could be expanded to demonstrate that you plan to describe how these models are implemented also?

***Specific comments:***

**Title:**

 1. Consider capitalising 'role and scope' in the title for consistency.

**Abstract:**

*Methods*

 1. More appropriate to say will be "reported" in accordance with PRISMA guidelines.

*Conclusions*

 2. I'm not sure what you mean by 'terminology' - is this to determine naming conventions for the role, or terminology in relation to community connector models?

**Introduction:**

 1.  'Community connector' is sometimes capitalized, sometimes not - choose one for consistency.

 2. In Paragraph 3/4, you discuss that there is "inconsistencies in their effectiveness" for community connectors. I think it is more appropriate to say that there is inconsistent *evidence* for effectiveness - as the literature is limited it is difficult to conclude whether one model is more effective over the other. 

 3. Missing word in Paragraph 4: "outcome measures across *studies*"

**Methods:**

 1. You state that there is limited evidence for the community connector role. How did you determine this? JBI recommend doing a preliminary search for existing reviews and stating this was performed in the introduction - was this how you determined a lack of evidence?

 2. Typos: Scoping review *is *registered, and changes will be *reported *not amended.

 3. You need to state how you will define community connector models in eligibility criteria - perhaps you mean to use the definition of community connector models in the introduction but this needs to be made clear. Are there any specific exclusion criteria? You may consider a table outlining specific inclusion/exclusion criteria which would be helpful for the reader. 

**Information Sources**

 1. The URL for JBI is incorrect, and you have included URLs for some sources but not others. Also the Joanna Briggs Institute goes by JBI now, please correct throughout the manuscript. 

 2. How will you systematically search and screen grey literature? Godin et al. have published useful guidance: Refer to reference no. 1

**Selection of sources of evidence**

 1.  You do not describe how articles will be screened or by whom which is important for replicability. You need to describe how titles and abstracts, full texts etc. will be screened. 

**Data charting process**

 1. Typo - third review *er*

**Synthesis of results**

 1. The sentence "quantitative data..." paragraph 2 seems to be repeating the sentence "a descriptive numerical..." paragraph 1?

Is the study design appropriate for the research question?

Yes

Is the rationale for, and objectives of, the study clearly described?

Yes

Are sufficient details of the methods provided to allow replication by others?

Partly

Are the datasets clearly presented in a useable and accessible format?

Not applicable

Reviewer Expertise:

Social prescribing, scoping reviews, health promotion, physical activity

I confirm that I have read this submission and believe that I have an appropriate level of expertise to confirm that it is of an acceptable scientific standard, however I have significant reservations, as outlined above.

## References

[1] : Applying systematic review search methods to the grey literature: a case study examining guidelines for school-based breakfast programs in Canada. *Systematic Reviews* .2015;4(1) : 10.1186/s13643-015-0125-0 10.1186/s13643-015-0125-0 26494010 PMC4619264

# Reviewer report: Reviewer response for version 1

**DOI:** 10.21956/hrbopenres.15695.r51142

**Published:** 2025-11-17

**Authors:** Vivian Welch, Linda Manirambona

**Affiliations:** 1 School of Epidemiology and Public Health, University of Ottawa, Ottawa, ON, Canada; 2 Interdisciplinary School of Health Sciences, University of Ottawa Faculty Health Sciences (Ringgold ID: 70363), Ottawa, Ontario, Canada

Thank you for the opportunity to review this great work ; it represents an important contribution to the field of health care, community services and healthy ageing.

 The protocol is well written and clear. Please consider the comments below for improvements:

Terminology and Consistency

 Ensure consistency in the use of terms: *Community Connector model*, *CC*, or *community connector*. You may choose one and apply it throughout the document.

Introduction

**Second paragraph:** It appears that addressing social determinants of health or community-based interventions is focused only on older adults. You may wish to clarify that these approaches apply to the general population but that this study specifically focuses on older adults.

**Third paragraph:**

- In some programs or countries, *community connectors* can also be based in primary care settings,see Tierney et al., 2025: *Experiences of integrating social prescribing link workers into primary care in England:Bolting on,fitting in, or belonging? A realist evaluation.*
- Considering the ‘’internationally’’ use of terminology for ‘’community connectors’’, in some low and middle-income countries (LMICs), *community health workers (CHWs)* may also deliver early treatment as part of their mandate.You may wish to clarify and redefine the terms and scope accordingly (see: https://health-policy-systems.biomedcentral.com/articles/10.1186/s12961-021-00748-4)You may also find relevant insights with this article *Engaging Community Health Workers (CHWs) in Africa: Lessons from the Canadian Red Cross supported programs* and clarify who might meet the criteria for being considered a *community connector* in this present study.

Scope and Definitions

 Clarify how you plan or anticipate categorizing the types of outcomes (health, social, …)

Eligibility Criteria

 Indicate whether there are any restrictions regarding countries or languages.

Data Charting

 Specify whether you will use any software (e.g. *Covidence*) for screening studies and data extraction.

Search Strategy

 The second sentence appears to be cut in 2, please revise for clarity and completeness.

Data item

 Since the definition of *community connector* is unclear (as noted in the introduction), you may consider adding it as an item to collect data if included studies have attempted to provide the definition

 You may also consider collecting data (if possible) about instruments/tools used to measure the outcomes

Synthesis of Results

 Consider providing a chart (e.g: Prisma Chart?) summarizing included and excluded studies, along with reasons for exclusion.

Is the study design appropriate for the research question?

Yes

Is the rationale for, and objectives of, the study clearly described?

Yes

Are sufficient details of the methods provided to allow replication by others?

Yes

Are the datasets clearly presented in a useable and accessible format?

Yes

Reviewer Expertise:

Evidence synthesis,global health,health equity and healthy ageing.

We confirm that we have read this submission and believe that we have an appropriate level of expertise to confirm that it is of an acceptable scientific standard.

## References

[1] : Experiences of integrating social prescribing link workers into primary care in England — bolting on, fitting in, or belonging: a realist evaluation. *British Journal of General Practice* .2025;75(752) : 10.3399/BJGP.2024.0279 e195-e202 10.3399/BJGP.2024.0279 PMC1180041039317389

[2] : Engaging Community Health Workers (CHWs) in Africa: Lessons from the Canadian Red Cross supported programs. *PLOS Global Public Health* .2024;4(1) : 10.1371/journal.pgph.0002799 10.1371/journal.pgph.0002799 PMC1079605938236795

[3] : Community health workers at the dawn of a new era: 5. Roles and tasks. *Health Research Policy and Systems* .2021;19(S3) : 10.1186/s12961-021-00748-4 10.1186/s12961-021-00748-4 34641903 PMC8506082

# Response

**Published:** 2025-11-18

**Authors:** Danielle Manning

**Affiliations:** Allied Health, University of Limerick, Limerick, County Limerick, Ireland

Dear Vivian, 

 I wish to thank you for your constructive and insightful feedback. I have taken your comments on board and will adjust the protocol accordingly. Thank you again for taking the time to peer review this article. 

 Kind Regards, 

 Danielle Manning
